# Quantitative Viscosity Mapping Using Fluorescence Lifetime Measurements

**DOI:** 10.1007/s11249-016-0807-3

**Published:** 2016-12-30

**Authors:** J. Dench, N. Morgan, J. S. S. Wong

**Affiliations:** 1 Department of Mechanical Engineering, Imperial College London, London, SW7 2AZ UK; 2 Shell Global Solutions (UK) Ltd, Brabazon House, Threapwood Road, Manchester, M22 0RR UK

**Keywords:** High-pressure rheology, Viscosity, Elastohydrodynamic lubrication, Fluorescence lifetime, In situ, Molecular rotor

## Abstract

Lubricant viscosity is a key driver in both the tribological performance and energy efficiency of a lubricated contact. Elastohydrodynamic (EHD) lubrication produces very high pressures and shear rates, conditions hard to replicate using conventional rheometry. In situ rheological measurements within a typical contact are therefore important to investigate how a fluid behaves under such conditions. Molecular rotors provide such an opportunity to extract the local viscosity of a fluid under EHD lubrication. The validity of such an application is shown by comparing local viscosity measurements obtained using molecular rotors and fluorescence lifetime measurements, in a model EHD lubricant, with reference measurements using conventional rheometry techniques. The appropriateness of standard methods used in tribology for high-pressure rheometry (combining friction and film thickness measurements) has been verified when the flow of EHD lubricant is homogeneous and linear. A simple procedure for calibrating the fluorescence lifetime of molecular rotors at elevated pressure for viscosity measurements is proposed.

## Introduction

 Friction in fluid film lubricated contacts is heavily influenced by the viscosity of the lubricant. The lubricant viscosity is pressure and usually shear dependent. Under sufficiently high pressures, the lubrication regime is characterised by nearly parallel, elastically deformed surfaces and is dominated by the pressure–viscosity response of the lubricant. This lubrication regime is known as elastohydrodynamic (EHD) lubrication. The pressure in these contacts is spatially heterogeneous. This pressure heterogeneity means the lubricant may perform very differently in different regions of the contact. Such spatial variations in pressure may not be accurately predicted by Hertzian mechanics. An increase in pressure just upstream of the inlet region, which is crucial to the development of a lubricant film, is also generated due to elastic deformation of the surface [1]. The pressure distribution, hence the potential variations in local viscosity, in and around an EHD contact, makes the study of local viscosity important.

Conventional techniques that estimate lubricant viscosity under conditions representative of EHD lubrication conditions include friction measurements [2] and high-pressure rheometry [3, 4]. Both techniques provide only the average viscosity of a lubricant film. When using friction measurements to determine the lubricant viscosity in a ball on flat contact, the lubricant is entrained and sheared to produce conditions close to those typically found, in engineering contacts. During these tests, the ball is half-submerged in an oil bath [5]. As a result, the measured friction contains contributions from both the actual friction generated in the contact and the viscous drag/churning moment experienced by the ball [6]. The latter may be significant when the actual friction force is low compared to the drag force. While the drag force can be estimated, it may render friction measurements, hence viscosity estimation, inaccurate under certain circumstances.

When using high-pressure rheometry techniques such as rotating Couette rheometers, a fixed sample volume is exposed to shear and pressure with the maximum shear rate usually around 10^4^ s^−1^ [7]. This is lower than shear rates commonly encountered in EHD contacts [8]. This technique also does not take into account the effect of the flow of the entrained lubricant. The nature of the technique makes it difficult to reach the required stress and strain levels without large increases in temperature [9]. Capillary flow viscometers can reach higher pressures and shear rates although they cannot reach high shear stresses. Falling cylinder viscometers can reach high pressures but have very low shear rates [7]. Therefore, these techniques are also difficult to apply to conditions similar to those found in EHD lubrication.

The difficulties of using conventional methods to obtain information about the lubricant viscosity in an EHD contact have prompted us to explore alternative ways. Ideally, the new method should allow measurements to be performed in an EHD contact close to typical operating conditions. It should also be able to capture local viscosity variations (if they exist) in and around the contact with good spatial resolution. Information on local viscosity is particularly valuable for understanding the origin of friction and the importance of surface effects (e.g. surface modifications and texturing).

Recent techniques to study in situ the viscosity of fluids in confinement such as atomic force microscopy [10] provide only a single average measurement representing properties of the whole fluid film. Techniques such as ultrasonic shear reflection [11] are limited by spatial resolution. However, recent improvements through the use of the matching layer method mean that where high spatial resolution is not an requirement, then this technique can be used to measure the viscosity in thin films [12]. Spectroscopic techniques provide the best approach for high spatial resolution measurements. Raman scattering for pressure sensing provides high spatial resolution (limited by beam size) across a wide range of pressures (up to 1.4 GPa) [13]. It therefore provides some information for local viscosity estimations if the pressure–viscosity relationship is known. However, as the viscosity of the fluid is not measured directly, the technique is incapable of detecting shear thinning. Phosphorescent nanoparticles have been shown to be sensitive to viscosity; however, information on their sensitivity and applicable range is not available [14]. The sensitivity of the nanoparticles photoluminescence to temperature also adds complexity to the measurements. Fluorescence recovery after photobleaching (FRAP) can be used to determine local fluid viscosity directly in static systems through measurement of diffusion [15]. It has also been applied to EHD contacts to obtain local through-thickness velocity profiles [16], and thus local shear rates, of the lubricant with high spatial resolution. At higher entrainment speeds more akin to typical engineering contacts, phosphorescence imaging has also been used [17]. In both cases, however, the local shear stress is required to determine the local viscosity. An attempt to obtain lubricant viscosity by coupling local velocity profile measurements and local shear stress measurements has been made [18]. However, only an average measurement for the whole contact far from most engineering conditions was obtained.

Fluorescence lifetime measurements using molecular rotor type fluorescent probes are frequently used in the biophysics community to measure the viscosity within cells [19, 20] and to study the growth of amyloid fibrils [21]. This technique has been recently adapted and applied to probe local lubricant viscosity in EHD contacts with microscale spatial precision [22]. However, quantitative measurements were only achieved in glycerol.

In this paper, a molecular rotor thioflavin-T is used to obtain the local viscosity distribution of the polymer IGEPAL CO-520 in a ball on flat EHD contact. These results are compared to high-pressure bulk viscosity measurements to validate the use of molecular rotors as local viscosity sensors for lubricants in the EHD regime. One of the characteristics of the EHD contact, the increase in pressure at the inlet critical in generating the fluid film, will also be visualised. The application of the technique to determine the shear stress across the contact will be demonstrated.

## Experimental

### Materials

Thioflavin-T (T3516) and IGEPAL CO-520 (238643) from Sigma-Aldrich were used as received. They are referred to as ThT and IGEPAL in this work, respectively. The properties of IGEPAL are shown in Table 1.

**Table 1:** Properties of IGEPAL CO520

Molecular weight (g/mol)	441
$\eta_{0}$ at 26 and 32 °C (Pa s)	0.23 and 0.17
$\alpha$ at 26 and 32 °C (GPa^−1^)	14.5 and 13.6

Viscosities and pressure–viscosity coefficients are from high-pressure rheometry (${\text{Shear stress}} < 40\;{\text{Pa}}$) and improved Yasutomi fits [23]

The model lubricant in this work consists of IGEPAL doped with 0.6–1.7 mM ThT. Details on its choice can be found in [22]. High dye concentration is required due to the ultra-thin films studied in this work and the dyes’ low quantum yield. ThT was dissolved in IGEPAL at 55 °C using a magnetic stirrer for one hour. The solutions were then filtered using a 1 μm filter (514-4027 Syringe filters, Acrodisc^®^, glass fibre VWR) to remove any undissolved impurities in the dye.

### Optically Accessible High-Pressure Rheometry

In this work, an optically accessible high-pressure rheometer based on a circular point contact is used [21]. This is created by a ball loaded on a flat disc. Both the speeds of ball and of the disc, $U_{\text{ball}}$ and $U_{\text{disc}}$, can be controlled independently to regulate the entrainment speed $U_{\text{e}}$ (Eq. 1), and the slide roll ratio SRR (Eq. 2) simultaneously. As $U_{\text{e}}$ increases, more lubricant is drawn into the contact, and the lubricant film increases in thickness.

1 $$U_{\text{e}} = (U_{\text{disc}} + U_{\text{ball}} )/2$$

2 $${\text{SRR}} = (U_{\text{disc}} + U_{\text{ball}} )/U_{\text{e}}$$

**Fig. 1:**
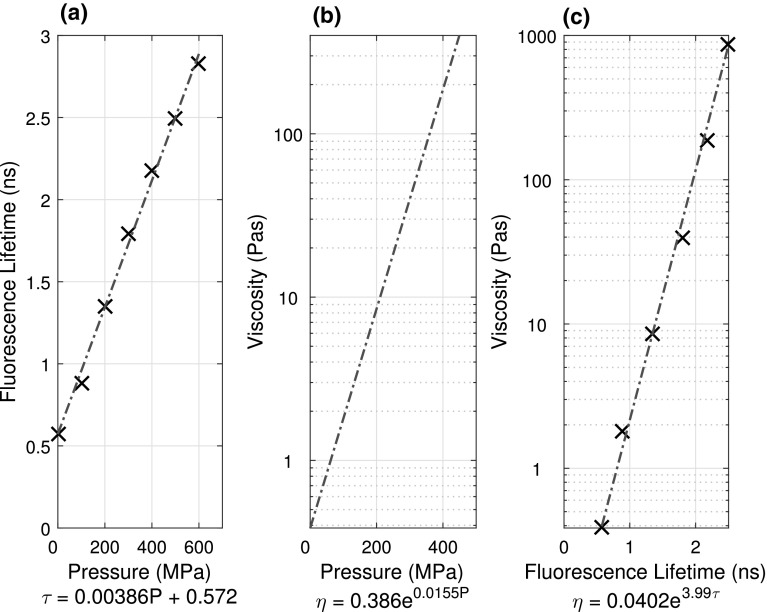
**a** Pressure–ThT lifetime relationship in IGEPAL from the high-pressure cell. **b** Pressure–viscosity relationship for IGEPAL. **c** Resulting calibration curve showing the relationship between ThT lifetime and the viscosity of IGEPAL

For an EHD point contact, its radius $a$ (Eq. 3) is controlled by the applied load $N$, effective radius $R$ and the combined elastic modulus $E^{*}$. The contact pressure varies with radial position $r$, and the pressure distribution $P\left( r \right)$ is commonly approximated by a Hertzian pressure distribution (Eq. 4). The maximum contact pressure $P_{max}$ occurs at the centre of the contact and is defined by Eq. 5, and the average pressure $P_{\text{a}}$ can be expressed by Eq. 6. The lubricant viscosity under EHD conditions affects both average and local shear stress within the contact. Viscosity and hence shear stress is a function of pressure, temperature and shear rate (for non-Newtonian liquids). The effect of pressure $P$ on viscosity $\eta$ can be described by the Barus relationship, where $\eta_{0}$ and $\alpha$ are the low pressure (bulk) viscosity and the pressure–viscosity coefficient, respectively (see Eq. 7). The Barus equation deviates from measurements at high pressure [24]. However, the peak pressures used in this work are less than 0.5 GPa. Hence, it is adequate in fitting our rheological data, as confirmed by the linearity of the pressure–fluorescence lifetime relationship for ThT in IGEPAL up to 600 MPa as shown in Fig. 1a.

3 $$a = \left( {\frac{3NR}{{4E^{*} }}} \right)^{{\frac{1}{3}}}$$

4 $$P\left( r \right) = P_{max} \left( {1 - \frac{{r^{2} }}{{a^{2} }}} \right)^{1/2}$$

5 $$P_{max} = \frac{3N}{{2\pi a^{2} }}$$

6 $$P_{\text{a}} = \frac{N}{{\pi a^{2} }}$$

7 $$\eta = \eta_{0} {\text{e}}^{\alpha P}$$

The point contact is chosen for this work as it allows the shear rate and the pressure to be controlled across the range of engineering interest. It also makes for easier comparison with results from friction and film thickness measurements. With independent control of entrainment speed and SRR, shear rates experienced by the lubricant film can be varied while maintaining a constant film thickness. This allows the effect of shear rate on both the local and average/apparent viscosity within the contact to be studied.

Transparent flat surfaces are used in this study to allow the optical access necessary for film thickness and fluorescence lifetime measurements. The surfaces chosen are a glass ball and a glass disc for rheological measurements. For film thickness measurements, where a reflective surface is required for interferometry, a steel (AISI52100) ball and chromium/silica coated glass disc are used. The properties of the rubbing surfaces, the range of contact pressures, shear rates and film thicknesses used are shown in Table 2. The contact pressures applied are chosen to prevent IGEPAL from shear thinning excessively.

**Table 2:** Experimental conditions

Average pressure (MPa)	180–280
Peak pressure (MPa)	270–420
Central film thickness (nm)	160–180
Entrainment speed unless otherwise stated (ms^−1^)	0.1
Shear rate (s^−1^)	2 × 10^4^–6 × 10^5^
E (GPa) and $\upsilon$ of AISI52100 steel	220 and 0.3
E (GPa) and $\upsilon$ of glass	70 and 0.2

To examine the local viscosity of an EHD lubricant in situ, fluorescence lifetime measurements are applied using the optically accessible point contact-based rheometer discussed above. The rheometer is mounted over an inverted microscope. The ball is loaded against the flat surface using a dead load. It is moved using an automated microscope stage which allows the local viscosity to be mapped with a high spatial resolution of 12 µm. Details of the set-up for fluorescence lifetime measurements are discussed in Sect. 2.3.2.

All tests were undertaken at an ambient temperature of 26 ± 1 °C, except the calibration which was undertaken at 21 °C. A SRR from 0 to 50% was applied. Fluorescence lifetime measurements show no correlation between SRR and viscosity. As the temperature rise in the contact depends on the SRR, at a given pressure, this indicates that the temperature rise due to shear heating in the present experiments is small, and its effect on the viscosity is within the statistical uncertainty of the measurements.

### Molecular Rotors and Fluorescence Lifetime Measurements

#### Brief Overview of ThT

The model lubricant in this work is ThT-doped IGEPAL CO-520. ThT is a molecular rotor which consists of a benzothiazole and an aminobenzene fragments joined together through a single bond allowing for intermolecular twisting [25]. When ThT is excited by light, the fragments twist relative to each other. The excited ThT has two routes to relaxation: (1) fluorescence emission and (2) a non-radiative twisted intermolecular charge transfer between the fluorescent LE (locally excited) state and the non-fluorescent TICT (twisted intermolecular charge transfer) state [26]. The latter involves the change in rotation angle between the two fragments [27]. This torsional relaxation process can be suppressed due to an increased fluid viscosity in the vicinity of ThT. This increases the probability of ThT fluorescence emission and leads to an increase in its quantum yield and its fluorescence lifetime [26]. This provides a direct relationship between quantum yield and viscosity [28]. An increase in ThT quantum yield of more than three orders of magnitude due to increased solvent viscosity has been observed [29], after which its quantum yield plateaus. ThT’s insensitivity to pressure and temperature makes it ideal to be used as a viscosity sensor in tribological contacts [30, 31]. The local viscosity of IGEPAL is thus extracted by measurements of ThT fluorescence lifetime at various positions in an EHD contact in this work.

#### Fluorescence Lifetime Measurements

Details of the fluorescence lifetime set-up used to measure ThT fluorescence lifetime in model lubricants can be found in [22] and is briefly described here. The optical rheometer described in Sect. 2.2 is placed over an inverted microscope. This allows the lubricant to be excited by 400 nm laser pulses (frequency doubled from 800 nm at a pulse width of 80 fs and a frequency of 80 MHz) through an objective (20X Olympus UPlanFL N, NA = 0.5). The power at the back of the microscope is less than 200 μW, and the focused beam size is 7 µm (FWHM). The emitted fluorescence is collected through the same objective, passes through emission filters, and is detected by a single-photon avalanche diode (SPAD). The instrument response of the SPAD is 80 ps FWHM. The fluorescence decay curve is then generated through time-correlated single-photon counting (TCSPC) using a Becker Hickl SPC-152 TCSPC card connected to the detector and the laser synchronisation output. The spatial resolution of the technique is based on the laser spot size (7 µm FWHM) and the spatial stability (5 µm). Therefore, the spatial resolution of the measurement is around 12 µm.

Calibration of the ThT lifetime in IGEPAL in static, bulk, conditions was conducted in a pressure cell at fixed pressure ranging from 0 to 600 MPa at 21 °C [22]. The same laser is directed into the cell through an optical window, and the fluorescence signal is collected at 90° through another window. The fluorescence was then passed through a focusing lens and emission filters before being collected on the SPAD. Results obtained, showing the relationship between ThT lifetime and applied pressure in static conditions, are presented in Fig. 1a.

### Analysis of Lifetime Decay Curves

Once ThT in IGEPAL is excited by the pulsed laser, the fluorescence intensity decays with time. The decay curves are fitted to find the characteristic decay time using a stretch exponential fit as shown in Eq. 8, where $I$ is the intensity at time $t$, $I_{0}$ is the intensity at $t = 0$, $\tau$ is the characteristic decay time (i.e. ThT fluorescence lifetime $\tau_{\text{ThT}}$) and $n$ is the stretch factor. A stretch exponential fit is commonly used to fit decay curves from molecular rotors [26, 31]. The stretch factor accounts for any other quenching mechanisms not listed in Sect. 2.3.1 [32]. In bulk conditions $n$ is between 0.68 and 0.75. As the solvent becomes more viscous and fluorescence emission becomes the dominant relaxation process, $n$ increases and tends to unity. For each decay curve, the maximum intensity value is identified and is taken as $I_{0}$. Data are then fitted from $t = 0.11 - 6.11\;{\text{ns}}$ after $I_{0}$.

8 $$I = I_{0} e^{{ - \left( {\frac{\tau }{t}} \right)^{n} }}$$

### Calibration, Rheological Characterisation and Choice of Operating Conditions

A pressure–viscosity relationship for IGEPAL-CO520 is needed to obtain the local viscosity of the lubricant in an EHD contact with fluorescence lifetime measurements. Bair measured the lubricant viscosity using a falling cylinder viscometer at elevated pressure at 20 and 25 °C [23]. A falling cylinder viscometer involves determining the terminal velocity of a sinker as it falls under gravity through the test fluid. The sinker calibration was carried out against values of Tris(2-ethylhexyl) trimellitate (TOTM) from the literature [33]. The improved Yasutomi model [34] was applied to estimate the viscosity of the working fluid at elevated pressures from 21 to 36 °C. These estimates are referred to as high-pressure rheological estimates in this work. For simplicity, the results of the model were fitted with the single exponential Barus equation within the experimental applied pressure range. The pressure–viscosity relationship for IGEPAL at 21 °C is shown in Fig. 1b. Together with the pressure–ThT fluorescence lifetime relationship we had obtained with the pressure cell [22] (Fig. 1a), a relationship between IGEPAL viscosity $\eta_{\text{IGEPAL}}$ and ThT fluorescence lifetime $\tau_{\text{ThT}}$ is established (Fig. 1c). It is fitted as a mono-exponential relationship and is used to estimate the local viscosity of lubricant in the EHD regime.

To determine if the link between Fig. 1a and b is valid in a contact as well as in the pressure cell, it is required that the lubricant is Newtonian. Previous results [22] and friction measurements (presented in Sect. 4) suggest that shear effects on IGEPAL are small for peak pressures below 400 MPa and slide roll ratios below 50% for an entrainment speed of 100 mms^−1^, within which the test conditions of this work fell. Note, once a molecular rotor fluorescence lifetime–lubricant viscosity relationship is established, the relationship can be applied to obtain local viscosity in a contact even if shear thinning occurs [22].

### Viscosity Mapping

**Fig. 2:**
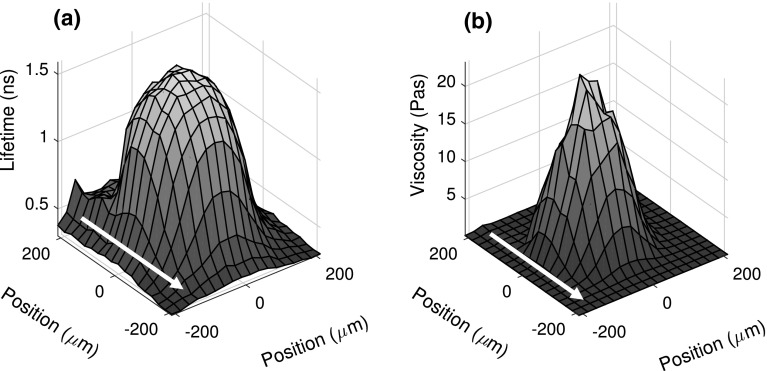
Maps obtained in contact at 380 MPa peak pressure under pure rolling, $h_{c}$ ≈ 170 nm. **a** ThT fluorescence lifetime and **b** IGEPAL viscosity distributions within the contact. Note that the conversion between ThT fluorescence lifetime and IGEPAL viscosity is based on Fig. 1. *Arrows* show flow direction

The local $\tau_{\text{ThT}}$ is measured across a region just larger than the contact using a grid size of 18 × 18 points (Fig. 2a). The $\tau_{\text{ThT}}$ map can be converted to a local $\eta_{\text{IGEPAL}}$ map using the calibration detailed in Sect. 2.5 and is shown in Fig. 2b. From Fig. 2b, the data points within Hertzian contact area are extracted. Data points within the contact are selected based on the calculated Hertzian contact area (Eq. 3) and a threshold lifetime value.

The centre of the contact was determined to find the contact area and to allow the calculation of the local pressure in the contact. The centre of the contact is identified as the point with the highest viscosity. This point is located based on a polynomial fit of suitably smoothed lifetime viscometry data. The contact area is then identified as a circular area around the centre thus located. This estimate of the contact area agrees well with the size of the experimental contacts observed using optical interference. This gives confidence that the actual contact pressure is the same as the Hertzian pressure. Local pressure can then be estimated with Hertzian pressure distribution (Eq. 4). Thus, the spatial distributions of $\tau_{\text{ThT}}$ in a contact allow us to relate local $\eta_{\text{IGEPAL}}$ to local pressure. Note smoothing of $\eta_{\text{IGEPAL}}$ data is performed only to find the centre of the contact. No smoothing procedure has been applied for results reported in this work.

**Fig. 3:**
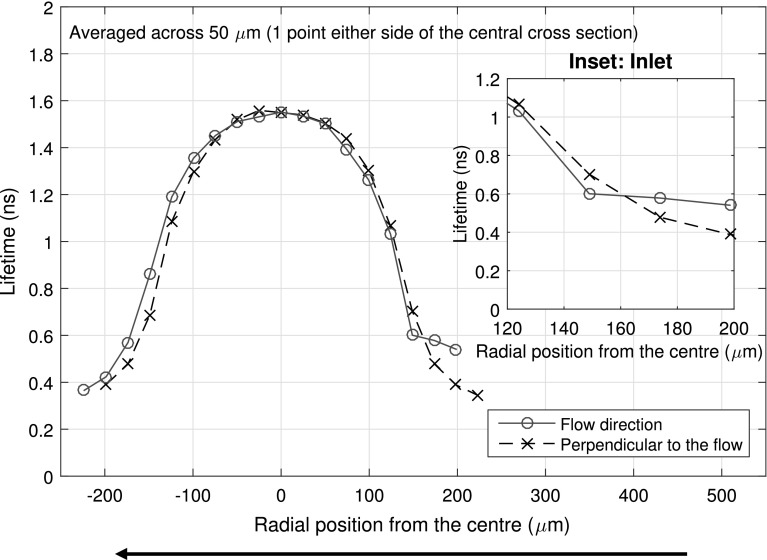
Cross section of local IGEPAL $\tau_{\text{ThT}}$ profile for a 380 MPa peak pressure contact (in pure rolling, $h_{c}$ ≈ 170 nm) both parallel and orthogonal to the flow direction. *Arrow* indicates the direction of flow. *Inset* shows the increase in $\tau_{\text{ThT}}$ at the inlet compared to the side edges of the contact

The local $\tau_{\text{ThT}}$ distribution as shown in Fig. 2a shows an increase in $\tau_{\text{ThT}}$ at the inlet of the contact. This is consistent with the inlet pressure rise commonly observed in an EHD contact. This inlet pressure rise region can extend up to half the contact width behind the contact and to a pressure of up to 150 MPa [9]. The corresponding viscosity rise at the inlet is better illustrated by plotting local $\tau_{\text{ThT}}$ along the parallel and orthogonal directions to the flow, passing through the centre of the contact, as shown in Fig. 3. It shows that $\tau_{\text{ThT}}$ at the inlet (solid line with circles, for position ≥170 μm) is approximately 0.2 ns higher than the rest of the bulk. This corresponds to an increase in viscosity from approximately 0.2–0.4 Pa s and is higher than the viscosity at the outlet and outside the contact orthogonal to the flow direction (dash line with crosses for position ≥170 μm). This suggests that the pressure at the inlet is about 50–100 MPa. Note Fig. 3 is an average across 3 grid points, so the value obtained is an underestimate of the actual $\tau_{\text{ThT}}$, due to the circular nature of the contact.

### Estimation of Average EHD Lubricant Viscosity

Average EHD lubricant viscosity is commonly estimated using friction measurements combined with film thickness measurements. Friction is measured using a PCS Instruments Mini Traction Machine (MTM2). This friction data are used to determine the average shear stress $\sigma$. The MTM2 measures the traction force $T$, and from this and the applied normal load $N$, the traction coefficient $\mu$ is calculated. Along with the contact radius, the shear stress can be calculated as shown in Eq. 9.

9 $$\sigma = \frac{\mu N}{{\pi a^{2} }}$$

**Fig. 4:**
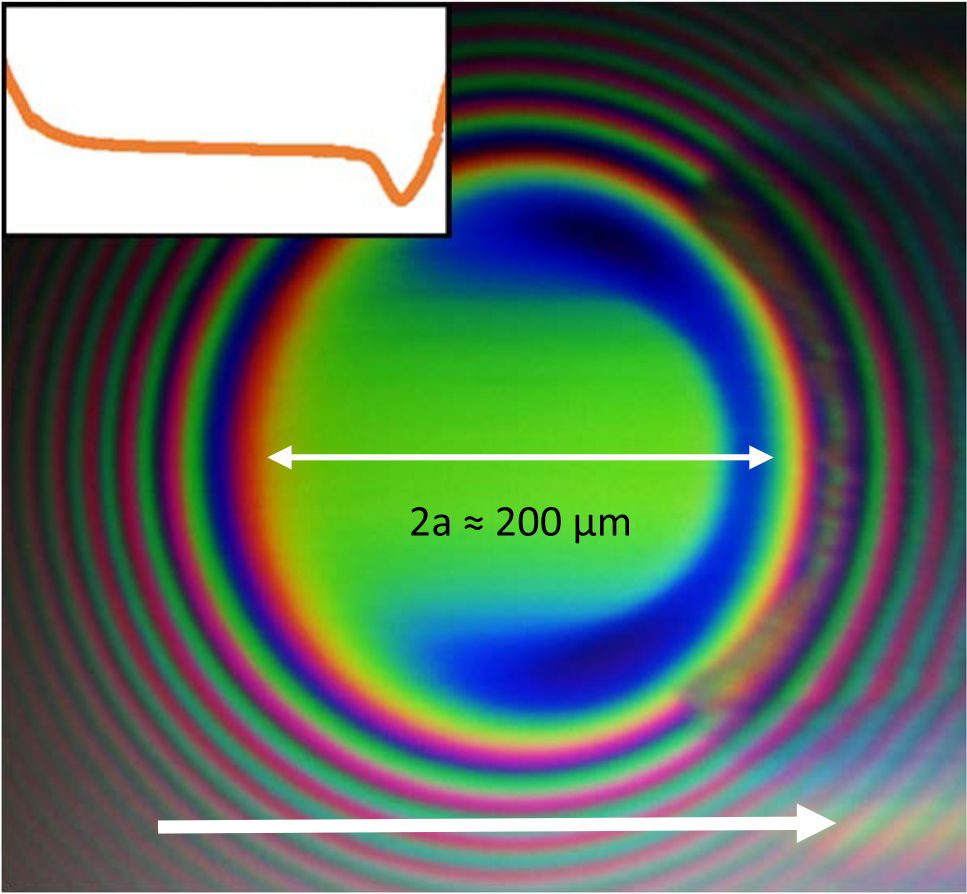
$U_{\text{e}}$ = 110 mm/s, 30% SRR, 370 MPa peak pressure, glass–steel SLIM (spacer layer imaging) image with optical interferometry. Central film thickness is approximately 170 nm, and the minimum film thickness is approximately 110 nm. The *inset* shows the film thickness profile in the flow direction (indicated by the *arrow*)

Optical interferometry [35] is used to measure the film thickness of the working fluid using a PCS Instruments EHD2 ultra-thin film measurement system. SLIM (spacer layer imaging method) [36] is used to determine the degree of homogeneity of the film thickness throughout the contact (Fig. 4). Using a steel–glass contact, a relationship for IGEPAL film thickness as a function of entrainment speed is obtained. EHD central film thickness of a circular contact $h_{c}$ can also be estimated using the Hamrock–Dowson relationship, as shown in Eq. 10 [37] where $U$ is the dimensionless speed parameter, $G$ is the dimensionless material parameter and $W$ is the dimensionless load parameter. These parameters contain either test conditions (load or speed), material properties or both. The measured results from film thickness measurements match estimates from Eq. 10 well (not shown).

IGEPAL film thickness for the conditions used for the fluorescence lifetime and friction measurements can be estimated from results obtained with a glass–steel contact by film thickness measurements. To account for the material change and varying test loads in these two measurements, adjustments are made by dividing out material and test condition factors for film thickness measurements on both sides of Eq. 10 and then factoring in the correct values for fluorescence lifetime and friction measurements. Comparison between the estimated film thickness for glass–steel contact and glass–glass contact shows negligible difference. All quoted IGEPAL film thickness for fluorescence lifetime measurements are based on interferometry measurements with glass–steel contacts and adjusted for load and material properties as described. The shear rate $\dot{\gamma }$ is determined from this film thickness $h_{c}$, the entrainment speed $U_{e}$ and the percentage slide roll ratio SRR. This is shown in Eq. 11.

10 $$h_{c} = 1.9U^{0.67} G^{0.53} W^{ - 0.067}$$

11 $$\dot{\gamma } = \frac{{\left( {SRR} \right)U_{e} }}{{100h_{c} }}$$

12 $$\eta_{a} = \frac{\sigma }{{\dot{\gamma }}}$$

With the average shear stress and average shear rate obtained from friction and film thickness measurements, respectively, an average or apparent viscosity is calculated using Eq. 12.

 The average viscosity of the EHD lubricant can also be estimated using the viscosity map obtained with fluorescence lifetime measurements (see Sect. 2.6). The local $\eta_{\text{IGEPAL}}$ in a viscosity map is an average through-thickness viscosity value at a particular position in a contact. Assuming the local viscosity at every position contributes equally to the average viscosity of the contact, and $J$ is number of data obtained and these points distribute evenly within the contact (as in the case of Fig. 2), the average in-contact viscosity $\eta_{\text{a}}$ is then the mean of the local viscosity values obtained from the map (Eq. 13). Eq. 13 is valid if the film thickness, hence the shear rate, within the contact region is reasonably homogeneous. The homogeneity of the film thickness in the contact is verified with interferometry (Fig. 4). Average viscosities obtained from friction and fluorescence lifetime measurements are compared to high-pressure rheological measurements.

13 $$\eta_{\text{a}} = \frac{{\mathop \sum \nolimits_{i = 1}^{J} Ae^{{B\tau_{i} }} }}{J}$$

## Results and Discussion

### Viscosity from Fluorescence Lifetime Measurements Compared with High-Pressure Rheology

**Fig. 5:**
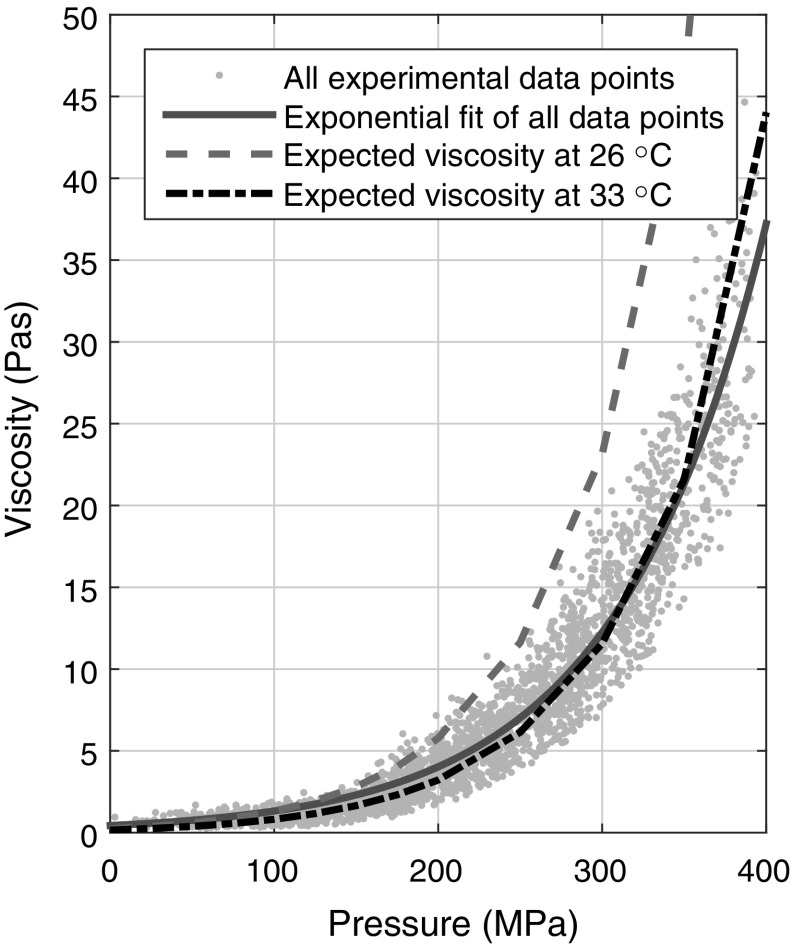
Local IGEPAL viscosity obtained across all SRR tested, showing the spread of data. Rheological fits at 26 (ambient temperature) and 33 °C (estimated average lubricant temperature at the inlet) shown for reference. Deviations from the rheological fit at 33 °C are likely due to variations in experimental temperatures

**Fig. 6:**
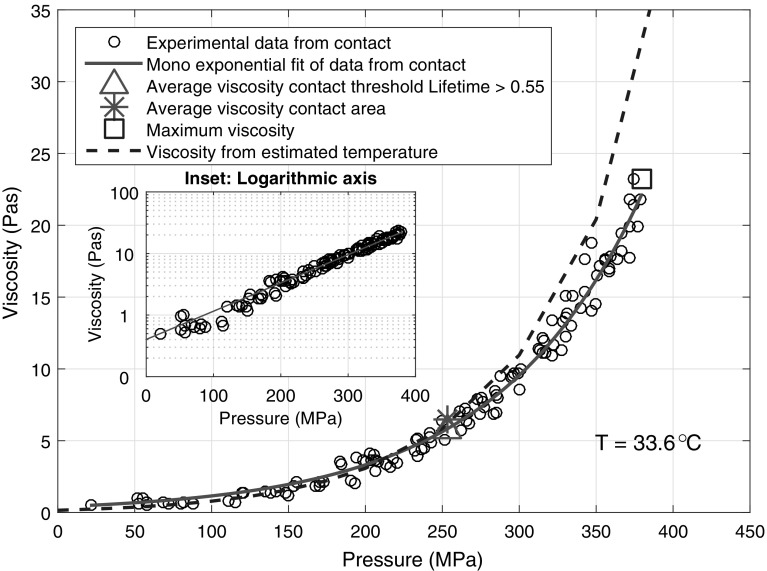
Local pressure versus viscosity for IGEPAL CO520 under pure rolling at a peak pressure of 380 MPa, *h* ≈ 170 nm. The *inset* shows this on a log-linear plot. Average viscosity from a lifetime threshold and based on the contact area are also displayed as a *triangle* and an *asterisk*, respectively. The maximum viscosity obtained at the maximum pressure is the *square*. The *dash line* is viscosity estimates based on contact temperature = 33.6 °C. The *red line* is the exponential fit through the experimental data points. This temperature is estimated by measuring the IGEPAL viscosity outside of the EHD contact (Color figure online)

By assuming a Hertzian pressure distribution, the relationship between local IGEPAL viscosity and local pressure can be obtained. Figure 5 shows the local $\eta_{\text{IGEPAL}}$ from 21 viscosity maps (324 data points each) taken from contacts with different peak pressures and SRR ranging from 0 to 50%. All the data collapses onto one master curve. This confirms that the effect of shear thinning is minimal across all test conditions. The scatter of the data points in the master curve is due to variations in temperature across experiments. The scatter in a single experimental set is actually low as can be seen in Fig. 6, supporting the assumption that the variation in temperature throughout each mapping experiment is small and that the change in experimental temperature is over a relatively long timescale. The relationship can be described by a mono-exponential fit (solid line, Fig. 5), suggesting it follows a Barus-type pressure–viscosity relationship. This is expected as the fluid viscosity can reasonably be estimated by the Barus equation in this pressure range and minimal shear thinning is expected in all of our experimental conditions. The result is, however, non-trivial. Should local shear thinning occur, which is promoted by high pressure, one would expect deviations to occur at high pressures.

Results in Fig. 5 are below the expected values at a testing temperature of 26 °C as based on high-pressure rheological estimates (dashed line, Fig. 5). They can be accounted for by a temperature range of approximately 29–36 °C. On average the results are best fitted by expected viscosity at 33 °C, suggesting either (a) shear thinning has occurred giving rise to lower than expected viscosity or (b) the contact was hotter than the ambient temperature. Since shear thinning is negligible, except potentially at the centre of the contact where pressure is high, in our study, this discrepancy is likely to be due to contact temperature rise from two possible sources. Firstly, there could be shear heating. Shear heating is more prevalent at high SRR and high pressure. As the data obtained from different pressures and SRR collapses onto one master curve, shear heating is unlikely to be significant. The second possibility is another heat source affecting temperature in the contact. It has been found that the bearing where the transparent disc sits heats up significantly as it rotates due to frictional heating within the bearing. Thus, the disc may heat up as a result. To confirm this for individual data sets, ThT fluorescence lifetime in IGEPAL around the contact (excluding the inlet) was averaged and used to estimate the average low pressure viscosity and hence the temperature of IGEPAL before entrainment. Results of one of the individual data sets are shown in Fig. 6. The estimated IGEPAL temperature before entrainment for this particular test was about 34 °C. As shown in Fig. 6, this set of data fits high-pressure rheological estimates at 33.6 °C. It thus confirms that while shear heating is negligible in the contact, the lubricant is hotter than expected before it was entrained into the contact and temperature variations across tests may give rise to the spread of the data in Fig. 5. From Fig. 6, it can be seen that for local pressures greater than approximately 280 MPa localised shear thinning occurs. This is due to errors in surface speeds causing shear thinning at high-pressure locations. Note with respect to a point contact, absolute pure rolling is very difficult to achieve as small errors in surface speeds can lead to significant shear rates. For example, 1% SRR under our conditions would provide a shear rate of approximately 6 × 10^3^ s^−1^. This will be discussed in detail in Sect. 4.2.

### Validation of the Approach to Use Average Measurements in an EHD Contact to Determine Lubricant Rheology

Since all data sets collapse into one master curve (see Fig. 5), for clarity the remaining discussion will be presented by using data obtained at a peak pressure of 380 MPa and 0 SRR (Fig. 6). The observation discussed below applies to all data set in this work.

The relationship between local IGEPAL viscosity and local pressure based on a single viscosity map (shown in Fig. 2b) is shown in Fig. 6. The inset in Fig. 6 shows the same data on a log-linear scale. The relationship can be described by a mono-exponential fit as discussed earlier (solid line, Fig. 6). Hence, the local viscosity of the fluid can be estimated by using a Hertzian pressure distribution and the Barus equation.

The average viscosity of the EHD lubricant is estimated based on ThT lifetime measurements as described in Sect. 2.7. The contact area was estimated in two ways (see Sect. 2.6). The average viscosity calculated with both area estimates (triangle and asterisk, Fig. 6) gives similar results. One would expect that the average viscosity and average pressure are related directly. In fact that is how average or nominal viscosity is calculated from a friction test, with an average shear stress calculated from the coefficient of friction. Plotting estimated average viscosity against average pressure Fig. 6 (asterisk and triangle respectively) shows they fall on the same exponential relationship fitted for the local viscosity data (circles). This observation supports the assumption mentioned in Sect. 2.7 that the thickness of the lubricant film, i.e. shear rate, in our EHD contact is homogeneous. More importantly, it suggests that the local velocity profiles within the contact obey Couette flow and are uniform. Under this condition, average measurements, such as friction measurements, can potentially be used to characterise the average viscosity of EHD lubricants. The validity of the homogeneity assumption can be assessed by studying the distribution of film thickness. The majority of the film will usually be made up by a flat central region; however, it is best to verify this using SLIM imaging. Similar procedures have been applied to estimate average IGEPAL viscosity for tests conducted at different peak pressures, SRR and entrainment speeds. Taking into consideration temperature variations among tests, average viscosities estimated at various average pressure from ThT fluorescence lifetime measurement match the relationship shown in Fig. 5 relatively well (not shown). This suggests that provided the homogeneity assumption holds, local viscosity measurements of this kind can be used to estimate average viscosity for a wide range of pressure with only a small number of experiments. This is particularly useful when the peak pressure that can be achieved by a point contact is higher than that can be achieved by conventional rheological techniques.

For conventional lubricants, they are commonly assumed to exhibit Couette flow in EHD contacts. In a contact where the flow profile is constant, should the EHD lubricant exhibit other types of flow such as Poiseuille’s flow, plug flow or shear banding, an apparent viscosity will be obtained from friction measurement, and it would not be equivalent to the average viscosity of the liquid. In cases where flow heterogeneity in an EHD contact is observed (see polybutene [16] and 5P4E [17]), using an average value across a contact may not be appropriate.

## Comparison with Friction Measurements

### Average Viscosity of EHD Lubricant

**Fig. 7:**
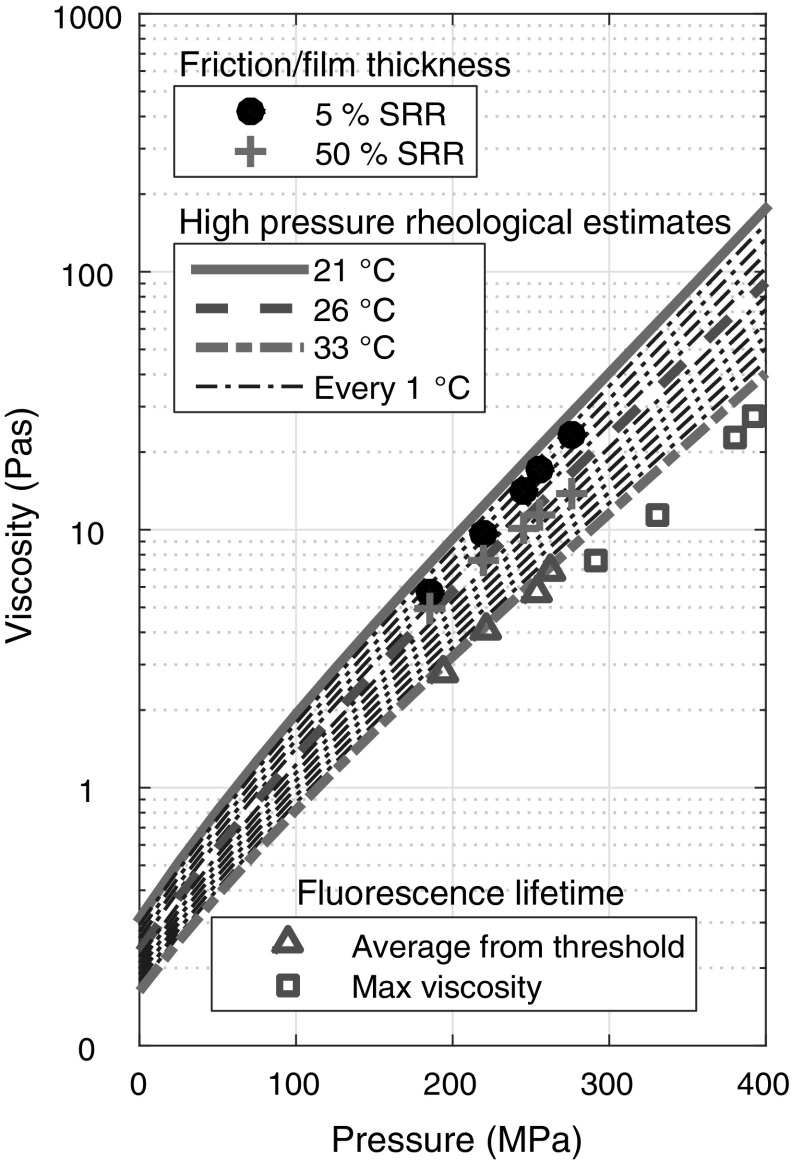
Comparison of viscosity estimates from combined friction and film thickness measurements (5 and 50% SRR average pressure of 185–276 MPa) at 26 °C, high-pressure rheological estimations at a range of temperatures between 21 and 33 °C and Fluorescence lifetime measurements under pure rolling conditions (4 tests undertaken on the same day)

The average viscosity of IGEPAL obtained from fluorescence lifetime measurement shown in Fig. 6 supports that average measurements, such as the combination of film thickness and friction measurements can potentially be used to measure the average viscosity of an EHD lubricant. To check the validity of this combined approach, the average viscosity of IGEPAL is estimated at various average Hertzian pressures and SRR. The results obtained at average pressure of 185–276 MPa and for SRR = 5 and 50% are shown in Fig. 7 (circles and pluses, respectively). In comparison with expected viscosities from high-pressure rheological estimates, the estimated viscosity from combined friction and film thickness measurements at 26 °C and SRR = 5% matches the high-pressure rheological estimates at 23–24 °C. This is likely to be due to temperature fluctuations and results in approximately a 25% increase in viscosity. It should be noted at these temperatures small fluctuations in temperature can lead to large changes in viscosity (see Table 3) Also on Fig. 7, the average viscosity of IGEPAL obtained with ThT fluorescence lifetime measurements in a similar average pressure range and the same entrainment speed under pure rolling is also presented (triangles). They fit high-pressure rheological estimates at approximately 33 °C well. As discussed in Sect. 3.1, the higher than expected bulk IGEPAL temperature in ThT fluorescence lifetime measurements is the reason for the discrepancy in average IGEPAL viscosity obtained from combined friction/thickness measurements and ThT fluorescence lifetime measurements. The maximum viscosity (open squares) is also presented to show the slightly lower than expected viscosity at the centre of the contact which is likely to be due to a shear effect.

**Table 3:** Effect of temperature and pressure on the viscosity of IGEPAL CO-520, based on data from high-pressure rheometry (Shear stress < 40 Pa) and improved Yasutomi fits [23]

Temperature (°C)	24	26	28	30	32	34	36
Pressure (MPa)	Viscosity (Pa·s)						
0	0.26	0.23	0.21	0.19	0.17	0.15	0.14
100	1.53	1.32	1.14	1.00	0.87	0.77	0.68
200	6.93	5.78	4.85	4.10	3.49	2.98	2.57
300	28.74	23.14	18.79	15.38	12.69	10.55	8.83
400	117.29	90.93	71.24	56.37	45.03	36.28	29.48

### Possible Shear Thinning

Average viscosity measurements based on combined friction/film thickness measurements show a reduction in average viscosity as SRR (i.e. shear rate) increases from 5 (solid circles, Fig. 7) to 50% (pluses Fig. 7). In fact, a reduction in viscosity of less than 30% was seen when friction measurements were conducted at an average contact pressure below 250 MPa and that the viscosity where this shear thinning started is 15–20 Pa·s, after which it started to drop (results not shown). This is contrary to results from ThT fluorescence lifetime measurements where no obvious shear effect was detected for average viscosity under these test conditions (see Fig. 7). The different phenomena observed between the two experiments may be due to differences in actual contact temperature (see Sect. 3.1), which was up to around 10 °C. This means the bulk IGEPAL viscosity for the ThT fluorescence lifetime measurements is lower than that of friction measurements. As a result, a high normal pressure or a higher shear rate/SRR is required to reach the critical shear stress for shear thinning to occur in our fluorescence lifetime measurements. This is supported by results obtained above 325 MPa (shown in Fig. 5), where the experimental fit of the data (red solid line) starts to deviate from expected viscosity from high-pressure rheological estimates at 33 °C (dot dashed line). This suggests shear thinning at a local pressure above approximately 300 MPa during our ThT fluorescence lifetime measurements.

The ability to obtain local viscosity information from fluorescence lifetime measurements is highlighted in Fig. 7, where local shear thinning is observed. The triangles and squares in Fig. 7 correspond to the average viscosity and local maximum viscosity from 4 sets of experiments under pure rolling conditions. All average viscosities match well with the high-pressure rheological estimates, while all maximum viscosities obtained at position with maximum pressure are lower than expected. This local shear thinning at low shear rates at high-pressure locations is not apparent in combined friction and film thickness measurements as the average pressure was less than 280 MPa although significant shear thinning is observed at average pressure of about 300 MPa (not shown). As the maximum local pressure approaches 400 MPa, local shear thinning at even relatively low shear rates is expected. Hence, local viscosity measurements have successfully predicted conditions where shear thinning will occur despite the low average pressure used in these tests.

### Shear Stress Mapping

With viscosity being directly linked to shear stress by shear rate, fluorescence lifetime measurements provide an opportunity to map the local shear stress in the contact. If the film thickness is known, which can easily be measured using optical interferometry or estimated using the Hamrock–Dowson equation, the shear rate can be calculated by Eq. 10. Local shear stress can then be estimated by simply multiplying the local viscosity by the shear rate (see Eq. 11).

**Fig. 8:**
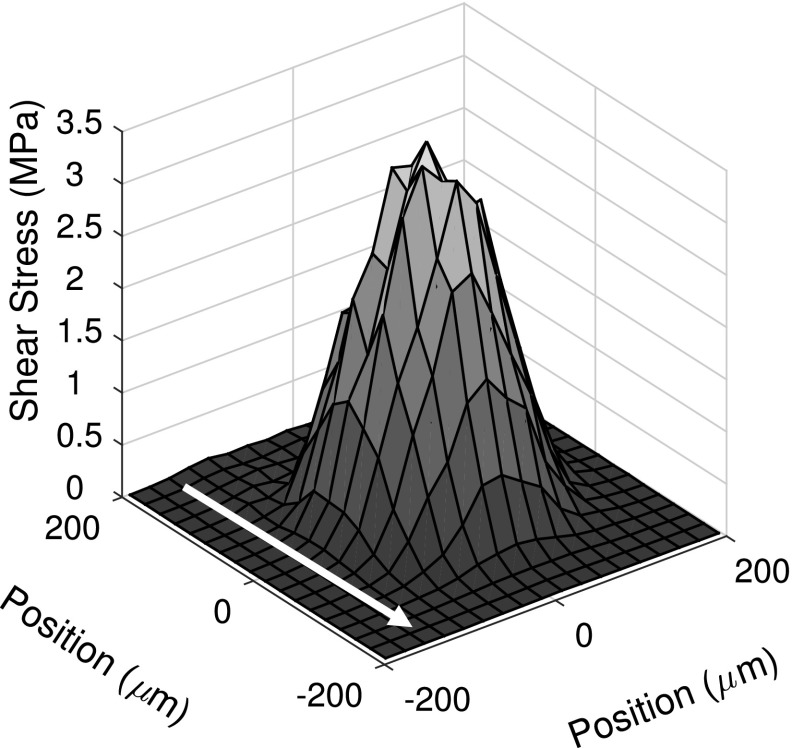
Shear stress map for a 342 MPa peak pressure contact at 30% SRR (1.771 × 10^5^ s^−1^), *h* ≈ 165 nm. Arrow shows the flow direction

The shear stress map of an EHD contact at a peak (average) pressure of 342 (230) MPa and shear rate of 1.71 × 10^5^ s^−1^ is shown in Fig. 8. In this case, the maximum (average) shear stress is 3.5 (2.3) MPa. From friction measurements under the similar conditions (30% SRR point used), the maximum (average) shear stress was 2.4 (1.6), 3.4 (2.3) and 4.1 (2.7) MPa at peak (average) pressure of 328 (218 MPa), 367 MPa (244 MPa) and 384 MPa (256 MPa), respectively. Using an exponential fit, the peak (average) shear stress at peak (average) pressure of 342 (230) MPa is estimated to be 2.7 (1.8) MPa. The discrepancy between the two estimates is likely due to differences in test temperatures. As discussed in Sect. 3.1, the temperature for fluorescence lifetime measurement was higher than that for friction measurements. This means the viscosity for the former tests was lower than that of the latter (see Fig. 7). The consequence, as described in 4.2, is that significant shear thinning occurs during friction measurements under these conditions, while during fluorescence lifetime measurements IGEPAL behaves close to Newtonian (see Fig. 7). The amount of shear thinning may be sufficient to render a lower shear stress in friction experiments. The need for interpolation also further increases the complexity of comparing the two measurements, where the pressure and temperature are not the same.

## Novel Calibration Method to Obtain the Fluorescence Lifetime–Viscosity Relationship of Molecular Rotors at High Pressure

**Fig. 9:**
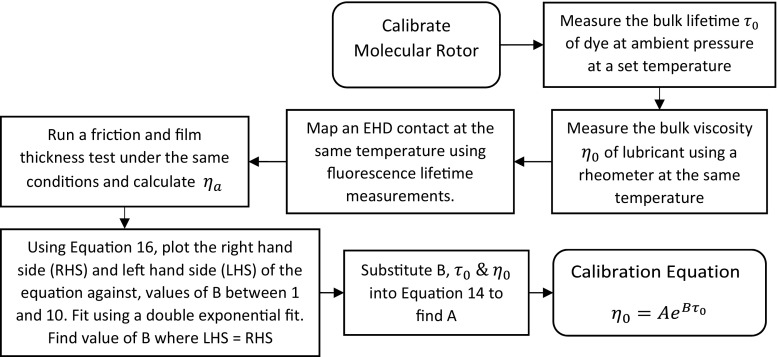
Flow chart on how to calibrate the fluorescence lifetime–viscosity relationship of a molecular rotor in a lubricant without a pressure cell

In this work, ThT fluorescence lifetime–pressure calibration at high pressure (see Fig. 1a) was conducted with an optically accessible high-pressure cell, in order to obtain the IGEPAL viscosity–ThT fluorescence lifetime calibration (Fig. 1c). Such a procedure is difficult as specialist equipment is required. Based on discussion in Sects. 4.1 and 4.2, we propose that a combined friction and film thickness measurements approach can be used to obtain the necessary rheological data for the relationship of fluorescence lifetime of a molecular rotor and viscosity of a lubricant, provided that the flow of the lubricant in the contact is homogeneous and linear. This calibration procedure is summarised in Fig. 9. It involves correlating results obtained at the same test conditions from fluorescence lifetime measurements with average viscosity of the lubricant obtained from combined friction–film thickness measurements. The key challenge is to ensure that all the tests are at the same temperature, so that the same viscosities are being compared across all tests.

Equation 14 shows the relationship between the bulk viscosity of the lubricant $\eta_{0}$ and the fluorescence lifetime of the added molecular rotor $\tau_{0}$, both of which can be obtained by simple bulk measurement at ambient conditions. Equation 13 can be rearranged to Eq. 15 where $\eta_{a}$ is obtained from combined friction–film thickness measurements and $\tau_{i}$ are local molecular rotor fluorescence lifetimes obtained from fluorescence lifetime measurement. Dividing Eq. 14 with Eq. 15 gives Eq. 16. Using a range of values for *B*, in the sensible range (1–10), one can obtain numerically the value *B* for which Eq. 16 is satisfied. Once *B* is found, it can be substituted into Eq. 14 to find *A* and now this calibration can be used to estimate the local lubricant viscosity in an EHD contact. Note, due to ThT’s quantum yield being independent of temperature [31] and pressure [30], the calibration coefficients *A* and *B* are only dependent on the lubricant chemistry.

14 $$\eta_{0} = A{\text{e}}^{{B\tau_{0} }}$$

15 $$\eta_{a} J = \mathop \sum \nolimits A{\text{e}}^{{B\tau_{i} }}$$

16 $$\frac{{\eta_{0} }}{{\eta_{a} J}} = \frac{{{\text{e}}^{{B\tau_{0} }} }}{{\mathop \sum \nolimits {\text{e}}^{{B\tau_{i} }} }}$$

## Conclusion

This work demonstrates quantitative viscosity measurements in an EHD contact on the microscale. The work utilises fluorescence lifetime measurements, which are compared to viscosity measurements from both friction measurements and high-pressure rheological estimates.

Viscosity distributions of ThT-doped IGEPAL CO520 in an EHD contact have been examined quantitatively by measuring in situ local ThT fluorescence lifetimes. The relationship between local IGEPAL viscosity and local pressure can be described by a single exponential fit within the range studied. Average viscosities at various average pressures were estimated from these viscosity maps, and these also fell on the same single exponential relationship. These results were compared to rheological estimates based on high-pressure rheometry and Yasutomi fits. When a correction for the test temperature was made based on the out of contact bulk IGEPAL viscosity, a good agreement was found. This supports the use of average viscosity measurements in capturing ensemble properties of EHD lubricants when the flow of lubricant in the contact is homogeneous and linear. Indeed average viscosity at various average applied pressures have been obtained with combined friction and film thickness measurements where the above-stated condition was met. The results indeed compared well by high-pressure rheological estimates, where the difference could be accounted for by a 2 °C error in temperature. The homogeneity assumption can be verified by visualising the contact and measuring lubricant film thickness with SLIM.

In addition, the results in this work support the use of local viscosity measurements in a point EHD contact, as described in this work, as a viable way of obtaining the average viscosity of lubricants across a wide range of pressures, which might not be reached by conventional rheological measurements. Taking advantage of the pressure distribution in a point contact, measurements for a large range of pressures can be obtained with relatively few experiments provide the homogeneity assumption is met.

An EHD contact can also be used for calibrating the relationship between fluorescence lifetime of a molecular rotor and the viscosity of a lubricant using conventional tribological techniques and the procedure detailed. However, this requires good temperature control across all experiments. If this can be achieved, this would negate the need for high-pressure or diamond anvil cells, thus increasing the accessibility of this technique for further exploration within the tribological community.

## References

[1] Wedeven LD, Evans D, Cameron A (1971). Optical analysis of ball bearing starvation. J. Lubr. Technol..

[2] Evans CR, Johnson KL (1986). The rheological properties of elastohydrodynamic lubricants. Proc. Inst. Mech. Eng. Part C: J. Mech. Eng. Sci..

[3] Bair S (2001). Measurements of real non-Newtonian response for liquid lubricants under moderate pressures. Proc. Inst. Mech. Eng. Part J: J. Eng. Tribol..

[4] Bair S (2002). The high pressure rheology of some simple model hydrocarbons. Proc. Inst. Mech. Eng..

[5] Müller, M., Fan, J., Spikes, H.: Influence of polymethacrylate viscosity index improvers on friction and wear of lubricant formulations. In: SAE Technical Paper 2007-01-1985 (2007)

[6] Gupta, P.K.: Churning and drag losses. In: Advanced dynamics of rolling elements, pp. 100–105. Springer, New York (1984)

[7] Bair, S.: Recent developments in high-pressure rheology of lubricants. In: Dowson, D., Taylor, C., Childs, T., Dalmaz, G., (eds.) Tribology series, vol. 30, Lubricants and lubrication: proceedings of the 21st leeds-Lyon symposium on tribology, pp. 169–187. Elsevier (1995)

[8] Bair S, Qureshi F (2003). The high pressure rheology of polymer-oil solutions. Tribol. Int..

[9] Spikes H, Jie Z (2014). History, origins and prediction of elastohydrodynamic friction. Tribol. Lett..

[10] Ahmed N, Nino DF, Moy VT (2001). Measurement of solution viscosity by atomic force microscopy. Rev. Sci. Instrum..

[11] Kasolang S, Dwyer-Joyce RS (2008). Viscosity measurement in thin lubricant films using shear ultrasonic reflection. Proc. Inst. Mech. Eng. Part J: J. Eng. Tribol..

[12] Schirru M, Mills R, Dwyer-Joyce R, Smith O, Sutton M (2015). Viscosity measurement in a lubricant film using an ultrasonically resonating matching layer. Tribol. Lett..

[13] Jubault I, Mansot JL, Vergne P, Mazuyer D (2002). In-situ pressure measurements using Raman microspectroscopy in a rolling elastohydrodynamic contact. J. Tribol..

[14] Hamza H, Albahrani SMB, Guillot G, Maillard M, Philippon D, Vergne P, Bluet JM (2015). Temperature and viscosity effects on the photoluminescence properties of alkyl-capped silicon nanoparticles dispersed in nonpolar liquids. J. Phys. Chem. C.

[15] Chen Y, Lagerholm BC, Yang B, Jacobson K (2006). Methods to measure the lateral diffusion of membrane lipids and proteins. Methods.

[16] Ponjavic A, Chennaoui M, Wong JSS (2013). Through-thickness velocity profile measurements in an elastohydrodynamic contact. Tribol. Lett..

[17] Galmiche B, Ponjavic A, Wong JSS (2016). Flow measurements of a polyphenyl ether oil in an elastohydrodynamic contact. J. Phys.: Condens. Matter.

[18] Fu L, Favier D, Charitat T, Gauthier C, Rubin A (2016). A new tribological experimental setup to study confined and sheared monolayers. Rev. Sci. Instrum..

[19] Kuimova MK (2012). Mapping viscosity in cells using molecular rotors. Phys. Chem. Chem. Phys..

[20] Kuimova MK, Yahioglu G, Levitt JA, Suhling K (2008). Molecular rotor measures viscosity of live cells via fluorescence lifetime Imaging. J. Am. Chem. Soc..

[21] Ban T, Hamada D, Hasegawa K, Naiki H, Goto Y (2003). Direct observation of amyloid fibril growth monitored by thioflavin T fluorescence. J. Biol. Chem..

[22] Ponjavic A, Dench J, Morgan N, Wong JSS (2015). In situ viscosity measurement of confined liquids. RSC Adv..

[23] Bair, S.: Private communication (2016)

[24] Sargent LB (1983). Pressure-viscosity coefficients of liquid lubricants. A S L E Trans..

[25] Erez Y, Liu YH, Amdursky N, Huppert D (2011). Modeling the nonradiative decay rate of electronically excited Thioflavin T. J. Phys. Chem. A.

[26] Stsiapura VI, Maskevich AA, Kuzmitsky VA, Uversky VN, Kuznetsova IM, Turoverov KK (2008). Thioflavin T as a molecular rotor: fluorescent properties of thioflavin T in solvents with different viscosity. J. Phys. Chem. B.

[27] Haidekker MA, Nipper M, Mustafic A, Lichlyter D, Dakanali M, Theodorakis EA, Demchenko AP (2010). Dyes with segmental mobility: molecular rotors. Advanced fluorescence reporters in chemistry and biology I: fundamentals and molecular design.

[28] Akers W (2004). A molecular rotor as viscosity sensor in aqueous colloid solutions. J. Biomech. Eng..

[29] Kuznetsova IM, Sulatskaya AI, Maskevich AA, Uversky VN, Turoverov KK (2016). High fluorescence anisotropy of Thioflavin T in aqueous solution resulting from its molecular rotor nature. Anal. Chem..

[30] Amdursky N, Gepshtein R, Erez Y, Koifman N, Huppert D (2011). Pressure effect on the nonradiative process of thioflavin-T. J. Phys. Chem. A.

[31] Amdursky N, Gepshtein R, Erez Y, Huppert D (2011). Temperature dependence of the fluorescence properties of thioflavin-T in propanol, a glass-forming liquid. J. Phys. Chem. A.

[32] Lee KC, Siegel J, Webb SE, Lévêque-Fort S, Cole MJ, Jones R, Dowling K, Lever MJ, French PM (2001). Application of the stretched exponential function to fluorescence lifetime imaging. Biophys. J..

[33] Diogo JCF, Avelino HMNT, Caetano FJP, Fareleira JMNA (2014). Tris(2-Ethylhexyl) trimellitate (TOTM) a potential reference fluid for high viscosity. Part II: density measurements at temperatures from (293 to 373) K and pressures up to 68 MPa. Fluid Phase Equilib..

[34] Bair S, Mary C, Bouscharain N, Vergne P (2013). An improved Yasutomi correlation for viscosity at high pressure. Proc. Inst. Mech. Eng. Part J: J. Eng. Tribol..

[35] Glovnea RP, Forrest AK, Olver AV, Spikes HA (2003). Measurement of sub-nanometer lubricant films using ultra-thin film interferometry. Tribol. Lett..

[36] Cann PM, Spikes HA, Hutchinson J (1996). The development of a spacer layer imaging method (SLIM) for mapping elastohydrodynamic contacts. Tribol. Trans..

[37] Hamrock BJ, Dowson D (1977). Isothermal elastohydrodynamic lubrication of point contacts: part III—fully flooded results. J. Lubr. Technol..

